# Intimate partner violence during pregnancy in Vietnam: prevalence, risk factors and the role of social support

**DOI:** 10.1080/16549716.2019.1638052

**Published:** 2019-07-22

**Authors:** Thanh Hoang Nguyen, Toan Van Ngo, Vung Dang Nguyen, Hinh Duc Nguyen, Hanh Thi Thuy Nguyen, Tine Gammeltoft, Dan Wolf Meyrowitsch, Vibeke Rasch

**Affiliations:** a Hanoi Medical University, Hanoi, Vietnam; b University of Copenhagen, Copenhagen, Denmark; c Departments of Obstetrics and Gynecology, Odense University Hospital, Odense, Denmark; d Departments of Clinical Research, University of Southern Denmark, Odense, Denmark

**Keywords:** Gender and Health Inequality, Intimate partner violence, pregnancy, prevalence, risk factors, social support

## Abstract

**Background**: Violence against women is a global public health problem. A better understanding of risk factors for intimate partner violence (IPV) exposure during pregnancy is important to develop interventions for supporting women being exposed to IPV.

**Objective**: The purpose of this study was to measure the prevalence of IPV during pregnancy and analyse how social support and various risk factors are associated with IPV.

**Methods**: A cross-sectional study conducted among 1309 pregnant women in Dong Anh district, Vietnam. Information about socio-economic conditions and previous exposure to IPV was collected when women attended antenatal care before the 24th gestational week. Information about social support information and exposure to IPV during pregnancy was collected in the 30^th^-34^th^ gestational week. Multivariable regression was used to identify associations between IPV, social support and other potential risk factors.

**Results**: The prevalence of IPV exposure during pregnancy was 35.2% (Emotional violence: 32.2%; physical violence: 3.5% and sexual violence: 9.9%). There was a statistically significant association between previous IPV exposure, lack of social support and IPV exposure during pregnancy. After adjustment for socioeconomic characteristics, pregnant women who had previously been exposed to IPV were more likely to be exposed IPV at least one time (AOR = 6.3; 95% CI: 4.9–8.2) as well as multiple times (AOR = 6.0; 95% CI: 4.5–8.0). Similarly, pregnant women having a lack of social support had a higher likelihood of being exposed to IPV at least one time (AOR = 3.1; 95% CI: 2.4–3.9) or multiple times (AOR = 2.9; 95% CI: 2.2–3.8).

**Conclusion**: IPV is relatively high during pregnancy in Vietnam. Previous exposure to IPV and lack of social support is associated with increased risk of violence exposure among pregnant women in Vietnam.

## Background

Intimate partner violence (IPV) against women is a global public health crisis [1]. World Health Organization (WHO) reported that 35% of women worldwide experienced physical and/or sexual violence in 2013, of which the rate of IPV has been generally higher in low and middle-income countries in comparison with high-income countries [2,3]. IPV rates are considerably high among women who are young [4–7]; unemployed [4,6–8]; have a low educational level and low income [4,7,9]; and live in rural areas [4,5,8,9]. In addition, those being married to a partner who is unemployed have low education or abuse alcohol are also at increased risk of IPV [4,5,9]. Pregnant women are particularly vulnerable to IPV. Although it is controversial whether IPV increases during pregnancy or not [10], this serious issue significantly reduces the health of women and their new-born children, as well as increases risk of infant mortality [11–14]. In low- and middle-income countries, the prevalence of pregnancy-related IPV range from 2%-57% [4,15].

Social support is an important instrument to prevent the occurrence of IPV as well as its adverse effects on pregnant women [16–19]. Social support is a critical function of social relationships, referring that a person has friends, family or social organizations who help providing a broader focus and positive self-image if needed [20]. It includes emotional (empathy, love, trust), instrumental (being available if need), and informational (supportive advice and suggestions) perspectives [20,21]. Prior studies suggested that women with high level of social support had a lower risk of IPV [22,23]. However, most of the studies have been conducted in high-income countries and not looked into the effects of different types of social support on the occurrence of IPV.

In Vietnam, according to a WHO’s report in 2014, the rate of pregnant women accessing antenatal care (ANC) is high with 94% due to a substantial progress in developing the primary health-care sector [24]. Nonetheless, a national study on violence against women in 2010 showed that 58% of women suffered violence at least once in their life including emotional (54%), physical (32%) and sexual (10%) violence [25]. This survey also indicated that 5% of pregnant women experienced physical violence [25]. However, literature about the relationship between social support and IPV is still limited. Therefore, this study aimed to measure the prevalence of IPV during pregnancy and analyse how social support and various risk factors are associated with IPV. The findings will partly contribute to design contextualized interventions and policies in addressing the IPV and its negative effects among pregnant women in Vietnam.

## Methods

### Study design and setting

A cross-sectional study was implemented that was nested within a larger cohort study involving 1337 pregnant women in Dong Anh District, Hanoi, Vietnam. The purposes of cohort study were to identify the prevalence of IPV during pregnancy and to measure the associations between IPV and the risk of low birth weight and preterm birth among pregnant women.

### Sampling procedure

**Figure 1.**
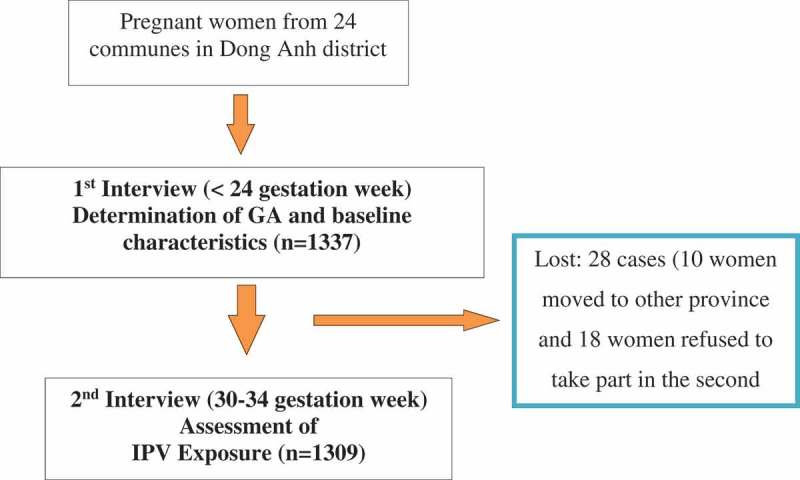
Follow-up chart.

Participants were pregnant women who were before 24 weeks of gestation (confirmed by ultrasound scanning) at Dong Anh District from May 2014 to August 2015. Data collection was conducted in three stages: (i) at enrolment or baseline (collecting socio-demographic and reproductive health information); (ii) at 30–34 weeks of pregnancy (collecting violence exposure information – before and during pregnancy, and social support); and (iii) at delivery (measuring birth weight and gestational age of the child). This study used data at stage (i) and (ii). There were 1.337 pregnant women enrolling in the study at stage (i) and 1309 (97.9%) completing surveys at stage (ii). The processing of data collection is presented in Figure 1.

### Data collection and measurement

Six interviewers were selected among 24 staffs in the Dong Anh District Population Centre by the research group. They were aged 35–45, had a college degree and had experience in working with the community. The interviewers were trained about interview skills in two weeks. A questionnaire was developed based on the WHO questionnaire on health and violence against women, which was used in the National Study on Violence against Women in Vietnam in 2010 [25]. The questionnaire was piloted among 30 pregnant women living in study settings and revised before implementing data collection.

#### Dependent variable

*Exposure to IPV during pregnancy*: Our participants were asked about whether they experienced any specific violence by their husband during their current pregnancy, as well as the number of times of being exposed to different types of violence. Emotional violence referred to those being insulted, humiliated, intimidated or threatened to hurt her or someone she cared for. Meanwhile, physical violence was defined as being slapped, pushed, hit, kicked, chocked or threatened. Sexual violence referred to pregnant women who were physically forced to have sexual intercourse, and accepted due to fear of partner’s reaction﻿, or were forced to do a sexual act which she found degrading or humiliating. Women were considered being exposed to IPV during pregnancy if they exposed to any form of IPV at least once.

#### Independent variables

*Socioeconomic characteristics*: Information about age, educational level (below high school/high school/above high school) and occupation (White-collar worker/Blue-collar worker/Farmer/Other (housewives, small business …)). was collected. The socioeconomic index was measured by asking: ‘Does your household have television, landline phone, mobile phone, refrigerator, computer or bank account ?’. The answers were coded in two categories: yes = 1; no = 0. After calculating the asset index of household, a quintile variable was defined and coded into three groups.

*Previous exposure to IPV*: To explore the history of IPV exposure, pregnant women were asked following question: ‘In the past 12 months before pregnancy, 1) Has your partner done things to scare or intimidate you on purpose?’; 2) ‘Has your partner threatened to hurt you or someone you care for?’; 3) ‘Has your partner hit you, slapped you, or thrown something at you that could hurt you’; and, 4) ‘Has your partner forced you or pressured you to have sexual intercourse when you did not want to’. Previous emotional IPV was determined if participants answered ‘Yes’ for question 1 and 2. Moreover, physical and sexual IPV were defined if the answers of question 3 and 4 were ‘Yes’, respectively. Women confirming any of the four questions were considered to have been exposed to previous IPV.

##### Social support

Social support was assessed using seven questions about emotional, instrumental and informational support [20]. Each question had five options to respond: always, most of the time, some of the time, rarely and never, ranking from 5 to 1.

Emotional support was measured via two questions: 1) ‘Do you have someone whom you know that you can always trust?’; and 2) ‘Do you have someone with whom you can share your thoughts and worries?’. Pregnant women had emotional support if the total score was 8 or more.

Instrumental support was evaluated using four questions: 1) ‘Do you have someone who cares for you by making sure you get enough to eat?’; 2) ‘Do you have someone who helps you with daily tasks (shopping, cooking, childcare, transportation, etc.)?’; 3) ‘Do you have someone in your family who takes a positive interest in your antenatal care visits?’; and 4) ‘Do you have someone who can help you financially if you need it?’. Participants had instrumental support if they had a total score of 16 or more.

Finally, one question was used to define informational support: ‘Do you have someone who helps you if you need to make difficult decisions?’. A score of 4 or more was used to identify having informational support.

The total score of seven questions was calculated (max score 35), and having social support was determined if the total score was 28 or more. The cut-off point was identified based on the median score among women who were not exposed to IPV during pregnancy, which was validated elsewhere [26,27].

### Statistical analysis

Data (S1 File, S2 File) were cleaned and double data entry was implemented using EpiData version 3.1 by two researchers. STATA version 14 was used for statistical analysis. Descriptive statistics were used to summarize the prevalence of IPV and frequency of IPV episodes during pregnancy. The analysis compared pregnant women who reported having experienced ‘any IPV’ and ‘IPV repeatedly’ with those not suffering any violence during pregnancy. For the latter, we excluded those with only one exposure and kept women who had multiple exposures. To determine risk factors associated with IPV exposure during pregnancy, crude odds ratios (OR) with 95% confidence intervals were calculated. Multivariable logistic regression then was used for controlling potential confounding factors (women’s age, education, occupation, economic status, previous IPV and social support). Statistical significance was considered at p value of less than 0.05.

### Ethical consideration

Our participants were informed about the study and written informed consents were then obtained. If they were less than 18 years old, the researcher explained to their parents about the study and the consent forms were signed by both women and their parents. After enrolment, participants were assigned different identification code (ID) and interviewers write the ID in each question sheet to instead of women’s identity. The title ‘Survey on Women’s Health and Life Experiences’ was used in research documents to not reveal that the study was about violence, which did not put respondents and interviewers at risk. The interviewers had to sign in ‘security agreement’ form as part of the employment contract. For those suffering IPV, after the entire cohort, we referred them to the appropriate social and psychological professional supports that were available in the community according to the guideline of World Health Organization [28]. The study was approved by the Ethical Committee of Hanoi Medical University, Vietnam (Decision 137/HMU IRB dated 29 November 2013).

## Results

Among 1,337 pregnant women enrolling at baseline, 1309 (97.9%) attended the second interview at the gestational week 30th-34th. The mean age was 27 years (SD = 4.8), ranging from 17 to 47 years. There were 44% educated at high school level education or above and 33% employed. Among women who were lost to follow up, the mean age was 27.6 years (SD = 4.3), 54% were educated at high school level or above and 10.7% were employed.

Table 1 presents the prevalence of IPV during pregnancy, 35.2% had been exposed to IPV at least once, and of those exposed, 85.9% had been exposed repeated times. Prevalence and frequency of the different types of IPV were: emotional violence: 32.2% (84.3% had been exposed 2 times or more); physical violence: 3.5% (38.1% had been exposed 2 times or more) and; sexual violence: 9.9% (92.3% had been exposed 2 times or more).

**Table 1.** The prevalence of IPV during pregnancy.

	n = 1309	%
**Any IPV during pregnancy (n = 461)**		
No IPV exposure	848	64.8
Exposure to any type of IPV	461	35.2
Once	65	14.1
2–5 times	325	70.5
More than 5 times	71	15.4
**Emotional IPV during pregnancy (n = 421)**		
No exposure emotional IPV	888	67.8
Exposure to emotional IPV	421	32.2
Once	66	15.7
2–5 times	303	72.0
More than 5 times	52	12.3
**Physical IPV during pregnancy (n = 46)**		
No exposure physical IPV	1263	96.5
Exposure to physical IPV	46	3.5
Once	28	60.9
2–5 times	15	32.6
More than 5 times	3	6.5
**Sexual IPV during pregnancy (n = 130)**		
No exposure sexual IPV	1179	90.1
Exposure to sexual IPV	130	9.9
Once	10	7.7
2–5 times	89	68.5
More than 5 times	31	23.8

Table 2 shows that in the univariate analysis, education level, occupation and economic status were found to be associated with IPV exposure. However, in multivariable regression, women who were workers and farmers were 1.9 (OR = 1.9; 95%CI: 1.4–2.7) and 2.4 times (OR = 2.4; 95%CI: 1.5–3.6) more likely to be exposed to IPV repeated times, respectively, which were higher compared to women being white-collar workers ư. In addition, those aged from 33 to 47 were less likely to suffer from repeated IPV (OR = 0.6; 95%CI = 0.4–0.9).

**Table 2.** Association between socio-economic characteristics and IPV during pregnancy.

		IPV exposure, any vs. no exposure			IPV exposure, repeated times vs. no exposure		
	Total n (%)	IPV exposure n (%) (n = 461)	Crude OR (95% CI)	AOR (95% CI)	IPV exposure n (%) (n = 396)	Crude OR (95% CI)	AOR (95% CI)
***Age groups***							
17–32	1122 (85.7)	406 (88.1)	1	1	349 (88.1)	1	1
33–47	187 (14.3)	55 (11.9)	0.7 (0.5–1.0)	0.6 (0.4–0.9)	47 (11.9)	0.7 (0.5–1.0)	0.6 (0.4–0.9)
***Education level***							
Less than high school	256 (19.6)	104 (22.6)	1	1	84 (21.2)	1	1
High school	482 (36.8)	175 (38.0)	0.8 (0.6–1.1)	0.9(0.6–1.2)	156 (39.4)	0.9 (0.7–1.3)	0.9 (0.7–1.3)
Above high school	571 (43.6)	182 (39.5)	0.7 (0.5–0.9)	0.9 (0.6–1.3)	156 (39.4)	0.7 (0.5–0.9)	0.9 (0.7–1.4)
***Occupation***							
White-collar worker	425 (32.5)	109 (32.5)	1	1	89 (22.5)	1	1
Blue-collar Worker	354 (27)	139 (30.2)	1.9 (1.4–2.6)	1.8 (1.3–2.5)	122 (30.8)	2.0 (1.5–2.8)	1.9 (1.4–2.7)
Farmer	170 (13)	74 (16.1)	2.2 (1.5–3.3)	2.2 (1.4–3.3)	64 (16.2)	2.4 (1.6–3.5)	2.4 (1.5–3.6)
Unemployed	360 (27.5)	139 (30.2)	1.8 (1.3–2.5)	1.8 (1.3–2.4)	121 (30.5)	1.9 (1.4–2.7)	1.9 (1.4–2.7)
***Economic status***							
Quintile 1	456 (34.8)	183 (39.7)	1	1	155 (39.1)	1	1
Quintile 2	657 (50.2)	218 (47.3)	0.7 (0.6–0.9)	0.8 (0.6–1.1)	192 (48.5)	0.8 (0.6–1)	0.9 (0.6–1.1)
Quintile 3	196 (15)	60 (13.0)	0.7 (0.5–0.9)	0.8 (0.5–1.2)	49 (12.4)	0.6 (0.4–0.9)	0.8 (0.5–1.1)

Table 3 indicates that after adjusting to age, education, occupation, and economic status, pregnant women being exposed to emotional, physical and sexual IPV previously, as well as not receiving emotional, instrumental and informational support had significantly high likelihood of being exposed to IPV during pregnancy. Experiencing previous emotional IPV had the largest effect on predicting multiple IPV during pregnancy (OR = 6.6; 95%CI = 5.1–8.7) compared to other factors. In addition, Crude OR and Adjusted OR in this table did not change significantly, showing stable relationships between previously experiencing IPV, social support and suffering IPV during pregnancy.

**Table 3.** Associate between act of previous violence and social support and IPV during pregnancy.

		IPV exposure, any vs. no exposure			IPV exposure, repeated times vs. no exposure		
	Total n (%)	IPV exposured n (%) (n = 461)	Crude OR (95% CI)	AOR* (95% CI)	IPV exposure n (%) (n = 396)	Crude OR (95% CI)	AOR* (95% CI)
**Previous emotional IPV**							
No	738 (56.4)	130 (28.2)	1	1	110 (27.8)	1	1
Yes	571 (43.6)	331 (71.8)	6.5 (4.9–8.5)	6.5 (5.0–8.5)	286 (72.2)	6.6 (4.9–8.8)	6.6 (5.1–8.7)
**Previous physical IPV**							
No	1160 (88.6)	364 (79.0)	1	1	312 (78.8)	1	1
Yes	149 (11.4)	97 (21.0)	4.1 (2.8–5.9)	4.0 (2.8–5.8)	84 (21.2)	4.1 (2.8–6)	4.1 (2.8–6)
**Previous sexual IPV**							
No	1200 (91.7)	393 (85.3)	1	1	339 (85.6)	1	1
Yes	109 (8.3)	68 (14.8)	3.4 (2.3–5.1)	3.1 (2.1–4.7)	57 (14.4)	3.3 (2.2–5.1)	2.9 (1.9–4.5)
**Emotional support**							
Yes	1010 (77.2)	308 (66.8)	1	1	311 (78.5)	1	1
No	299 (22.8)	153 (33.2)	2.4 (1.8–3.1)	2.2 (1.7–2.9)	85 (21.5)	2.5 (1.9–3.3)	2.2 (1.7–3.0)
**Instrumental support**							
Yes	759 (58)	199 (43.2)	1	1	173 (43.7)	1	1
No	550 (42)	262 (56.8)	2.6 (2.0–3.3)	2.5 (2.0–3.2)	223 (56.3)	2.5 (2–3.2)	2.4 (1.9–3.1)
**Informational support**							
Yes	933 (71.3)	286 (62.0)	1	1	248 (62.6)	1	1
No	376 (28.7)	175 (38.0)	1.8 (1.4–2.3)	2.2 (1.3–3.7)	148 (37.4)	1.9 (1.5–2.5)	1.8 (1.4–2.3)

*Adjusted for women’s age, education, occupation, economic status.

Table 4 shows that pregnant women who had previously been exposed to IPV were more likely to be exposed IPV at least one time (AOR = 6.3; 95% CI: 4.9–8.2) as well as multiple times (AOR = 6.0; 95% CI: 4.5–8.0). Similarly, pregnant women having a lack of social support had a higher likelihood of being exposed to IPV at least one time (AOR = 3.1; 95% CI: 2.4–3.9) or multiple times (AOR = 2.9; 95% CI: 2.2–3.8).

**Table 4.** Multiple logistic regressions between previous IPV/social support and IPV during pregnancy.

		IPV exposure, any vs. no exposure			IPV exposure, repeated times vs. no exposure		
	Total n (%)	IPV exposure n (%)	Crude OR (95% CI)	AOR* (95% CI)	IPV exposure n (%)	Crude OR (95% CI)	AOR* (95% CI)
**Previous IPV**							
No	702 (53.6)	119 (25.8)	1	1	100 (25.2)	1	1
Yes	607 (46.4)	342 (74.2)	6.3 (4.8–8.4)	6.3 (4.9–8.2)	296 (74.8)	6.5 (4.8–8.7)	6.0 (4.5–8.0)
**Social support**							
Yes	732 (55.9)	173 (37.5)	1	1	143 (36.1)	1	1
No	577 (44.1)	288 (62.5)	3.2 (2.5–4.1)	3.1 (2.4–3.9)	253 (63.9)	3.4 (2.6–4.4)	2.9 (2.2–3.8)

*Adjusted for women’s age, education, occupation, economic status

## Discussion

### Prevalence of IPV

In our study, more than one-third of pregnant women had been exposed to IPV during pregnancy with 32.2% women reporting exposure to emotional, 3.5% to physical and 10% to sexual violence. These findings were different from a previous work that the prevalence of emotional, physical and sexual violence was 28.4%, 13.8%, and 8.0%, respectively [9]. Our physical and sexual violence rates also differed from those in other countries such as China (11.9% ad 9.1%, respectively), and Japan (2.3% and 1%, correspondingly) [6,8]; and much lower compared to Thailand, where 54% of pregnant women were exposed to emotional violence, 27% to physical violence and 19% to sexual violence [29]. These dissimilarities might be explained by the use of different measures and definitions of violence [5,9,30,31]. In this study, we used the WHO questionnaire for measuring IPV among women, while the Chinese study used the revised conflict tactics scale and the Japanese study used an IPV screening tool (VAWS) [6,8]. In addition, the disparity may be due to the willingness of women to disclose the IPV exposure, which relies on the culture, the fear of retaliation and the availability of support [5,32,33]. In Vietnam, particularly in rural areas, the perceived social norm is that women were responsible for the marital harmony while men had a role as primary income earners (i.e. ‘pillars’ in the family) [34]. Therefore, domestic violence might be treated as private issues and could not be disclosed for a ‘Happy’ family [35–37].

### Risk factor of IPV

Our current study shows in multivariable regression that being blue-collar workers or farmers were risk factors of being exposed to IPV repeatedly, while those having higher age were less likely to suffer multiple IPV. This is consistent with two recent meta-analyses [4,9] and studies in other settings such as India, Bangladesh, Nepal, Japan, and Egypt [6,7,38]. In Vietnam, while white-collar workers were related to high education and income, those being blue-collar workers or farmers were more likely to have low education [39]. A possible explanation behind the found linkage between these social vulnerabilities (young, manual jobs such as blue-collar workers and farmers) and IPV is that those women had more difficult access to information about women’s rights. This situation may foster higher levels of gender inequality and greater acceptance of norms that support violence against women [4,9].

Our result demonstrates that previous IPV exposure was a strong risk factor for IPV during pregnancy, which was consistent with other previous studies [4,9]. Indeed, repeated IPV exposure is common among Vietnamese women and being pregnant does not protect women against violence [25]. Since the women were asked about IPV that had occurred during the past 12 months, we assumed that the abusive acts previously and during current pregnancy were enacted by the same partner. To stop the vicious cycle of repeated IPV exposure and prevent the negative impact of IPV among pregnant women, it is of major importance to start implementing IPV screening programs in relation to antenatal care. Globally, screening IPV, receiving counselling from, and building trusting relationships with health-care providers or other social workers would decline the occurrence of IPV during pregnancy and improve knowledge and capacities to cope with IPV among pregnant women [40,41].

### Social support

Social support was found to be a protective factor against IPV during pregnancy. This result is consistent with a study from Spain, which reported that women who were supported socially had a lower probability of IPV compared to those not being supported socially [22]. In literature, social support could maintain a positive self-esteem and self-concept [20], which might change women’s attitudes toward IPV and reduce risk of IPV [30]. It also reduced IPV-related eating disorders [17] and stress during pregnancy [16,42], assisting in preventing adverse effects of IPV on pregnant women’s mental health. However, it is cautious that the nature of cross-sectional design in these studies and our study as well does not allow determining the causal relationship between IPV and social support; thus, further longitudinal and experimental studies should be warranted to fill this knowledge gap.

## Limitation of the study

Our study has some important limitations that should be considered. First, it relies on a cross-sectional design; therefore, it is not possible to conclude the causal relationship between social support and IPV. Second, selection bias may occur since the study only included women attending antenatal care. However, the vast majority of women in the study setting attended the service, implying the representative of our sample to the pregnant women living in Dong Anh district. Third, because violence is a sensitive topic, it may be difficult to obtain valid information about IPV exposure. To overcome this drawback, the research assistants, who performed the interviews, developed a good relation with the women and the interviews were performed rather as conversations than strict questionnaire-based interviews. To create an atmosphere of confidentiality and empathy, the research assistants were further encouraged to share their own experiences with the women they interviewed. Finally, 28 pregnant women who were lost to follow-up might have different IPV and social support characteristics compared to those participating in the follow-up study, which could affect the results. Other limitation, when minors participated in the study, they required the consent of a guardian as their mother or father. It was maybe a social desirability bias. In Vietnam, the law stipulates that minors are people under 18 years old. When they participated in community studies, they required the consent of a guardian as their mother or father. In our study, to avoid the risk of being involved in the study, we changed the research name to “research on women’s health and life experiences“. In our study, there were 02 participants who were 17 years old. We have explained the purpose of the study to the mother who was their guardian and they agreed to sign the consent form. The information of interview was coded and without disclosure to anyone including their guardian.

## Conclusion

IPV is relatively high during pregnancy in Vietnam. Previous exposure to IPV and lack of social support is associated with increased risk of violence exposure among pregnant women in Vietnam.

## Paper context

Violence against women is a global problem. IPV during pregnancy does not only affect the women’s health but also imposes adverse health effect on the new-born children. A better understanding of risk factors for IPV exposure during pregnancy is important to develop interventions to support women. In this study (conducted among 1309 pregnant women), we show that there was a statistically significant association between previous IPV exposure, lack of social support and IPV exposure in pregnancy.

## References

[1] Garcia-Moreno C, Jansen HAFM, Ellsberg M, et al Prevalence of intimate partner violence: findings from the WHO multi-country study on women’s health and domestic violence. Lancet. 2006;368:1260–8. 17027732 10.1016/S0140-6736(06)69523-8

[2] World Health Organization Global status report on violence prevention. Geneva: World Health Organization; 2014.

[3] Garcia-Moreno C, Pallitto C, Devries K, et al Global and regional estimates of violence against women: prevalence and health effects of intimate partner violence and non-partner sexual violence. Geneva (Switzerland): World Health Organization; 2013.

[4] Shamu S, Abrahams N, Temmerman M, et al A systematic review of African studies on intimate partner violence against pregnant women: prevalence and risk factors. PLoS ONE. 2011;6:e17591 Epub 2011/ 03/17. 21408120 10.1371/journal.pone.0017591 PMC3050907

[5] Capaldi DM, Knoble NB, Shortt JW, et al A systematic review of risk factors for intimate partner violence. Partner Abuse. 2012;3:231–280. Epub 2012/ 07/04. 22754606 10.1891/1946-6560.3.2.231 PMC3384540

[6] Kita S, Yaeko K, Porter SE. Prevalence and risk factors of intimate partner violence among pregnant women in Japan. Health Care Women Int. 2014;35:442–457. Epub 2013/ 12/20. 24350998 10.1080/07399332.2013.857320

[7] Ibrahim Z, Fau - Sayed Ahmed WA, Sayed Ahmed W, et al Intimate partner violence among Egyptian pregnant women: incidence, risk factors, and adverse maternal and fetal outcomes. Clin Exp Obstet Gynecol. 2015;42:212–219. 26054122

[8] Chan KL, Brownridge DA, Tiwari A, et al Associating pregnancy with partner violence against Chinese women. J Interpers Violence. 2011;26:1478–1500. Epub 2010/ 05/25. 20495098 10.1177/0886260510369134

[9] James L, Brody D, Hamilton Z Risk factors for domestic violence during pregnancy: a meta-analytic review. Violence Vict. 2013;28:359–380. Epub 2013/ 07/19. 23862304 10.1891/0886-6708.vv-d-12-00034

[10] Jasinski JL Pregnancy and domestic violence: a review of the literature. Trauma Violence Abuse. 2004;5:47–64. Epub 2004/ 03/10. 15006296 10.1177/1524838003259322

[11] RE AL B, Bhutta Z, Caulfield LE, et al Maternal and child undernutrition: global and regional exposures and health consequences. Lancet. 2008;371:243–260. 18207566 10.1016/S0140-6736(07)61690-0

[12] Abadi MN, Ghazinour M, Nygren L, et al Birth weight, domestic violence, coping, social support, and mental health of young Iranian mothers in Tehran. J Nerv Ment Dis. 2013;201:602–608. Epub 2013/ 07/03. 23817159 10.1097/NMD.0b013e3182982b1d

[13] Chai J, Fink G, Kaaya S, et al Association between intimate partner violence and poor child growth: results from 42 demographic and health surveys. Bull World Health Organ. 2016;94:331–339. Epub 2016/ 05/06. 27147763 10.2471/BLT.15.152462 PMC4850526

[14] Hoang TN, Van TN, Gammeltoft T, et al Association between intimate partner violence during pregnancy and adverse pregnancy outcomes in Vietnam: a prospective cohort study. PLoS One. 2016;11:e0162844 Epub 2016/ 09/16. 27631968 10.1371/journal.pone.0162844 PMC5025080

[15] KM KS D, Johnson H, Stöckl H, et al Intimate partner violence during pregnancy: analysis of prevalence data from 19 countries. Reprod Health Matters. 2010;18:158–170. 21111360 10.1016/S0968-8080(10)36533-5

[16] Iranzad I, Bani S, Hasanpour S, et al Perceived social support and stress among pregnant women at health centers of Iran - Tabriz. J Caring Sci. 2014;3:287–295. Epub 2015/ 02/25. 25709981 10.5681/jcs.2014.031 PMC4333898

[17] Schirk DK, Lehman EB, Perry AN, et al The impact of social support on the risk of eating disorders in women exposed to intimate partner violence. Int J Women’s Health. 2015;7:919–931. Epub 2015/ 12/10. 26648759 10.2147/IJWH.S85359 PMC4664489

[18] Norbeck JS, DeJoseph JF, Smith RT A randomized trial of an empirically-derived social support intervention to prevent low birthweight among African American women. Soc Sci Med. 1996;43:947–954. Epub 1996/ 09/01. 8888464 10.1016/0277-9536(96)00003-2

[19] Hetherington E, Doktorchik C, Premji SS, et al Preterm birth and social support during pregnancy: a systematic review and meta-analysis. Paediatr Perinat Epidemiol. 2015;29:523–535. Epub 2015/ 09/04. 26332279 10.1111/ppe.12225

[20] Weber ML She stands alone: A review of the recent literature on women and social support. Canada: Prairie Women’s Health centre of Excellence; 1998.

[21] House JS Work stress and social support. Canada: Addison-Wesley Publicing Company; 1981.

[22] Plazaola-Castano J, Ruiz-Perez I, Montero-Pinar MI The protective role of social support and intimate partner violence. Gac Sanit. 2008;22:527–533. Epub 2008/ 12/17. 19080928 10.1016/s0213-9111(08)75350-0

[23] Branch KA The role of social support in the use of intimate partner violence: an exploration of the role of social support in heterosexual women’s use of non-lethal intimate partner violence. Saarbrücken, Germany: VDM Verlag Dr Muller Aktiengesellschaft; 2008.

[24] World Health Organization Sucess factors for women’s and children’s health: policy and programme highlights from 10 fast-track countries. Geneva, Switzerland: World Health Organization; 2014.

[25] Henrica AFMJ, Vung ND, Anh HT, et al Results from the national study on domestic violence against women in Vietnam. Vietnam: Ministry of Health; 2010.

[26] Sarason IG, Levine HM, Basham RB, et al Assessing social support: the social support questionnaire. J Pers Soc Psychol. 1983;44:127–139.

[27] Coker AL, Smith PH, Thompson MP, et al Social support protects against the negative effects of partner violence on mental health. J Womens Health Gend Based Med. 2002;11:465–476. Epub 2002/ 08/08. 12165164 10.1089/15246090260137644

[28] World Health Organization Responding to intimate partner violence and sexual violence against women: WHO clinical and policy guidelines. Italia: World Health Organization; 2013. 24354041

[29] Saito A, Creedy D, Cooke M, et al Effect of intimate partner violence on antenatal functional health status of childbearing women in Northeastern Thailand. Health Care Women Int. 2013;34:757–774. Epub 2013/ 06/25. 23790193 10.1080/07399332.2013.794459

[30] Trinh OT, Oh J, Choi S, et al Changes and socioeconomic factors associated with attitudes towards domestic violence among Vietnamese women aged 15–49: findings from the multiple indicator cluster surveys, 2006–2011. Glob Health Action. 2016;9:29577 Epub 2016/ 03/08. 26950567 10.3402/gha.v9.29577 PMC4780074

[31] Yount KM, VanderEnde K, Zureick-Brown S, et al Measuring attitudes about women’s recourse after exposure to intimate partner violence: the ATT-RECOURSE scale. J Interpers Violence. 2014;29:1579–1605. Epub 2013/ 12/26. 24368681 10.1177/0886260513511536

[32] Krug EG, Mercy JA, Dahlberg LL, et al The world report on violence and health. Lancet. 2002;360:1083–1088. Epub 2002/ 10/18. 12384003 10.1016/S0140-6736(02)11133-0

[33] Valladares E, Pena R, Persson LA, et al Violence against pregnant women: prevalence and characteristics. A population-based study in Nicaragua. BJOG. 2005;112:1243–1248. Epub 2005/ 08/17. 16101603 10.1111/j.1471-0528.2005.00621.x

[34] Nhị TT, Hạnh NTT, Gammeltoft TM Emotional violence and maternal mental health: a qualitative study among women in northern Vietnam BMC Women’s Health. 2018;18:58. 10.1186/s12905-018-0553-9 PMC5921269 29699557

[35] Vung ND, Ostergren PO, Krantz G Intimate partner violence against women in rural Vietnam – different socio-demographic factors are associated with different forms of violence: need for new intervention guidelines? BMC Public Health. 2008;8:55. 18267016 10.1186/1471-2458-8-55 PMC2275257

[36] Kwiatkowski L Domestic violence and the “Happy Family” in Northern Vietnam. Anthropol Now. 2011;3:20–28.

[37] Schuler SR, Lenzi R, Hoang T-A, et al Recourse seeking and intervention in the context of intimate partner violence in Vietnam: aqualitative study. J Family Issues. 2016;37:1151–1173.

[38] Anand E, Unisa S, Singh J Intimate partner violence and unintended pregnancy among adolescent and young adult married women in south Asia. J Biosoc Sci. 2017;49:206–221. Epub 2016/ 06/22. 10.1017/S0021932016000286 27324924

[39] Tran T, Tran A, Pham T, et al Local governance and occupational choice among young people: first evidence from Vietnam. Hanoi, Vietnam: University of Economics and Buisness, Vietnam National University, Hanoi; 2017.

[40] Nelson HD, Bougatsos C, Blazina I Screening women for intimate partner violence: a systematic review to update the U.S. preventive services task force recommendation. Ann Intern Med. 2012;156:796–808. 22565034 10.7326/0003-4819-156-11-201206050-00447

[41] Van Parys AS, Verhamme A, Temmerman M, Verstraelen H Intimate partner violence and pregnancy: a systematic review of interventions. PLoS One. 2014;9:e85084. 10.1371/journal.pone.0085084 PMC3901658 24482679

[42] Shishehgar S, Mahmoodi A, Dolatian M, et al The relationship of social support and quality of life with the level of stress in pregnant women using the PATH model. Iran Red Crescent Med J. 2013;15:560–565. Epub 2014/ 01/08. 24396574 10.5812/ircmj.12174 PMC3871742

